# Undiagnosed osteoid osteoma of the spine presenting as painful scoliosis from adolescence to adulthood: a case report

**DOI:** 10.1186/1748-7161-4-9

**Published:** 2009-04-27

**Authors:** George Sapkas, Nicolas E Efstathopoulos, Michael Papadakis

**Affiliations:** 1 1st Department of Orthopaedic Surgery, University of Athens, Attikon University Hospital, Haidari, Greece; 2 2nd Department of Orthopaedic Surgery, University of Athens, Agia Olga General Hospital, N. Ionia, Greece; 3 Spine Unit, Metropolitan Hospital, Faliro, Greece

## Abstract

Presented here is a case of a young woman, with an undiagnosed osteoid osteoma of the spine, which presented with painful scoliosis in adolescence and was treated by bracing until her accession to adulthood. A more thorough investigation, years after the initial one, revealed the tumor. Surgical excision and stabilization offered the long-awaited cure. Misdiagnosis resulted in intractable pain for years, deformity, the discomfort of brace therapy, and the frustration of a prolonged yet ineffective treatment.

## Background

The most common cause of painful scoliosis in adolescents is osteoid osteoma of the spine [1]. Up to 25% of all osteoid osteomas are found in the spine, of which 60% are located in the lumbar spine, 27% in the cervical, 12% in the thoracic and 2% on the sacrum [2]. There is a very strong correlation of this neoplasm with scoliosis, since two thirds of spinal osteoid osteomas manifest as painful scoliosis [2]. It is however extremely common for the wrong diagnosis to be initially set in a large, albeit unknown, number of cases [3]. The lesion is often recognized after months or years of ineffective bracing [4].

Presented here is a case of a young woman, who presented with painful scoliosis in adolescence and was treated by bracing until her accession to adulthood, without the osteoid osteoma she suffered from being diagnosed. She therefore endured intractable pain for years, on top of which was added the discomfort of brace therapy. The impact of a prolonged yet ineffective treatment on the psychological status of a patient with chronic pain as well as deformity should not be overlooked.

## Case presentation

The patient presented initially at the age of 15 years. Her main symptom was diffuse pain on the thoraco-lumbar spine. Radiographic examination at that time consisted only of plain X-rays. The only valuable finding was, reportedly, a left thoraco-lumbar scoliosis of 25 degrees. The treating physician, without any further work-up, recommended treatment with a brace. The patient complied with this treatment until the age of 21 years, without finding some relief from the pain that afflicted her.

**Figure 1:**
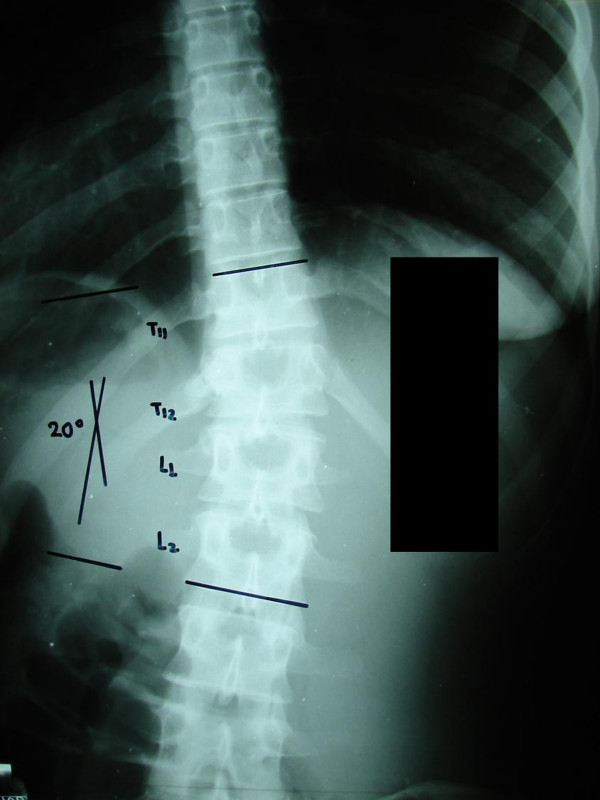
**Postero-anterior radiograph at age 21**. The tumour is indiscernible.

**Figure 2:**
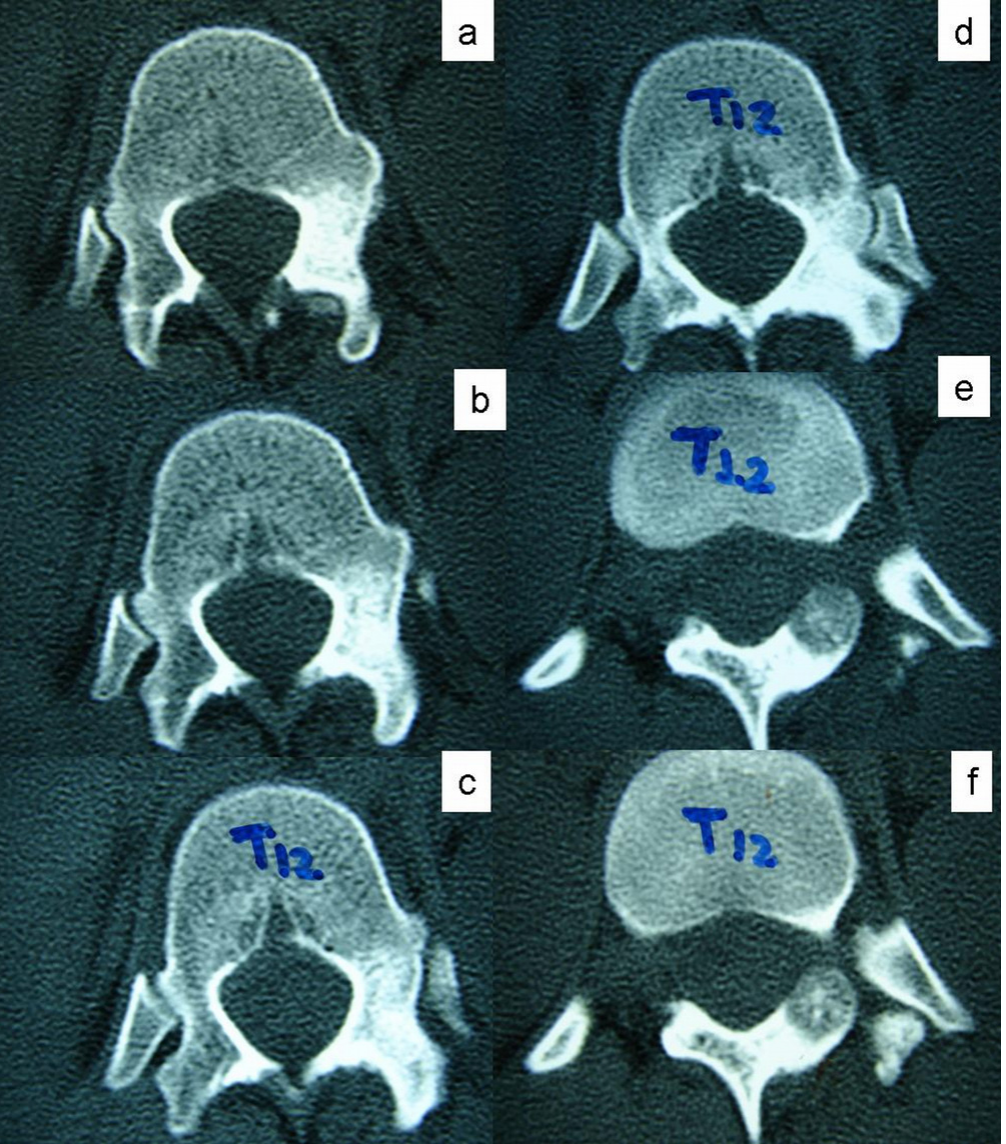
**Consecutive CT slices of the T12 vertebra**. The nidus can be seen at the pars interarticularis, with reactive sclerosis of the superior articular process and lamina and marrow oedema of the pedicle extending up to the vertebral body.

**Figure 3:**
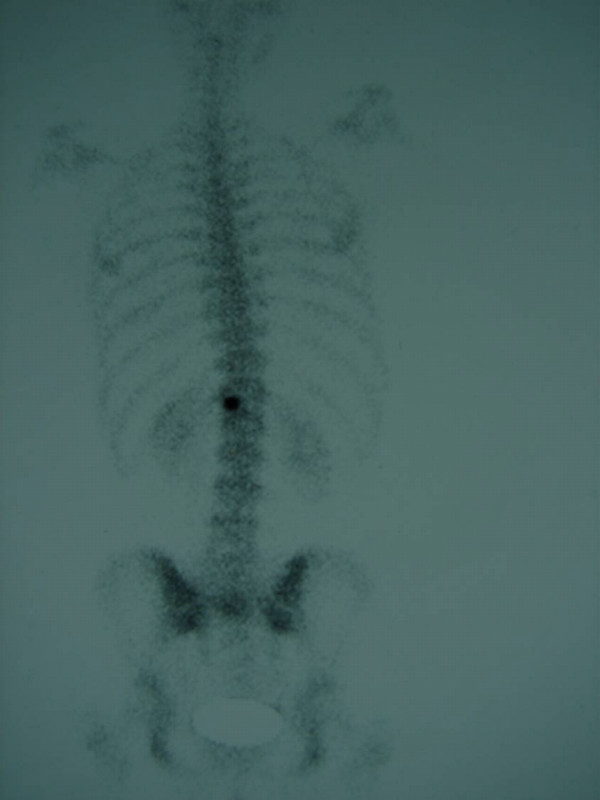
**Posterior view 99m-Tc MDP bone scan**. The only area of increased tracer uptake is the left portion of the T12 vertebra.

At the end of this course and with the symptoms still persisting, the patient decided to get a second opinion. Plain X rays showed that the scoliosis was now reduced to 20 degrees (figure 1). CT scanning of the spine revealed a tumor on the left posterior arch of T12, which affected the lamina, pars interarticularis and inferior facet process (figure 2). She was promptly subjected to a full radiologic examination, where no other lesions were found. Scintigraphy was positive only at the aforementioned location (figure 3).

Because of the involvement of the facet joint and the resultant instability that its excision would cause, it was decided that tumor removal should be complemented by stabilization. The patient underwent a posterior procedure, which consisted of left hemi-laminectomy, articular process included, curettage of the left pedicle up the vertebral body and posterior fixation with transpedicular screws and rods from T11 to L1. The fixation was augmented with heterologous bone graft only from the right (opposite to the tumor) side, and a screw was placed in the right T12 pedicle. Histological examination confirmed the lesion to be an osteoid osteoma.

**Figure 4:**
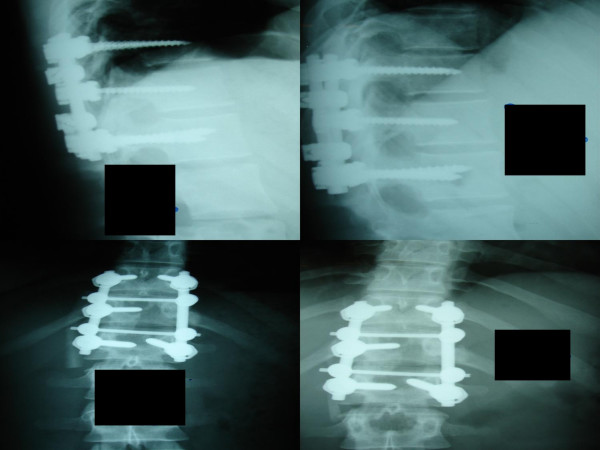
**Post-operative radiographs 1 month (left) and 1 year (right) after surgery**.

After an uneventful recovery, the patient was discharged with a clear improvement of her symptoms. Complete resolution was achieved shortly thereafter. On the latest follow up the patient remains asymptomatic, and now lives an active, unconstrained life (figure 4).

## Conclusion

The peak incidence of osteoid osteomas occurs in adolescence [2]. Back ache is an uncommon complaint in adolescents and young adults, and when it occurs in association with paravertebral muscle spasm and scoliosis, osteoid osteoma of the spine must be suspected [3]. Spinal radiographs often miss the tumor, due to their small size and complex anatomy of the spine [5]. Nevertheless, every patient with scoliosis should receive a thorough radiographic evaluation at presentation, especially so when back pain is also present. An MRI would have at least demonstrated some pathologic findings, if not the tumor itself, and would have prompted further diagnostic testing [6].

Spinal osteoid osteomas are treated surgically, and in the majority of cases complete removal is achieved by curettage [1]. Therefore, curettage should be the treatment of choice wherever possible, as this prevents iatrogenic instability and the need for a more extensive operation. Even though there have been reports of tumor recurrence in cases where curettage alone was performed, [7-9], their number is so small that the risks involved with major surgery do not offset the risk of recurrence. The patient described in this report had to undergo a more extensive operation, because of the involvement of the facet joint.

Timing of the operation is of great significance, and should be performed as quickly as possible. A demonstrative series is that of Ransford et al [10], where in almost all those patients whose symptoms had lasted for more than 15 months, scoliosis persisted despite removal of the tumor. Additionally, the majority of those patients exhibited vertebral rotation and the degree of scoliosis was related to the duration of symptoms.

There are reports in the literature of cases where the tumor subsides without surgery [11-13]. However, the risk of an antalgic scoliosis to be converted to a structural one is quite high. The pressure that develops on the end-plates from the concave side inhibits growth, while on the convex side growth continues unhindered. Thus, delayed treatment could result in structural scoliosis despite the excision of the tumor [10,14].

In conclusion, a case of spinal osteoid osteoma, unrecognized and untreated for many years, is described. Neither did the associated scoliosis become structural, nor did the tumor regress. In the end, surgical resection and stabilization offered the long-awaited cure. Years of suffering and frustration would have been avoided, had the diagnostic approach been the appropriate one.

## Consent

Written informed consent was obtained from the patient for publication of this case report and any accompanying images. A copy of the written consent is available for review by the Editor-in-Chief of this journal.

## Competing interests

The authors declare that they have no competing interests.

## Authors' contributions

GS, NE, and MP participated in the design of the study, analysis and writing of this manuscript. GS and NE participated also in revising critically the manuscript. All authors read and approved the final manuscript.
